# Adverse cardiovascular outcomes between insulin-treated and non-insulin treated diabetic patients after percutaneous coronary intervention: a systematic review and meta-analysis

**DOI:** 10.1186/s12933-015-0300-6

**Published:** 2015-10-07

**Authors:** Pravesh Kumar Bundhun, Nuo Li, Meng-Hua Chen

**Affiliations:** Institute of Cardiovascular Diseases, The First Affiliated Hospital of Guangxi Medical University, Nanning, Guangxi 530027 People’s Republic of China

**Keywords:** Cardiovascular outcomes, Type 2 diabetes mellitus, Percutaneous coronary intervention

## Abstract

**Background:**

Type 2 diabetes mellitus (DM) patients have worse adverse cardiovascular outcomes after Percutaneous Coronary Intervention (PCI). However, the adverse cardiovascular outcomes between insulin-treated and non-insulin treated DM patients have been a subject of debate. We sought to compare the short-term (<1 year) and long-term (≥1 year) cardiovascular outcomes between insulin-treated and non-insulin treated DM patients after PCI.

**Methods:**

Medline and Embase databases were searched for studies by typing ‘diabetes and percutaneous coronary intervention/PCI’ or ‘insulin-treated and non-insulin treated diabetes mellitus and PCI’. Endpoints included adverse cardiovascular outcomes reported in these DM patients during the corresponding follow-up periods. Odd Ratio (OR) with 95 % confidence interval (CI) was used to express the pooled effect on discontinuous variables and the pooled analyses were performed with RevMan 5.3.

**Results:**

21 studies have been included in this meta-analysis consisting of a total of 21,759 diabetic patients (6250 insulin-treated and 15,509 non-insulin treated DM patients). Short term mortality, myocardial infarction, target lesion revascularization, major adverse cardiac effects and, stent thrombosis were significantly higher in insulin-treated diabetic patients (OR 1.69, 95 % CI 1.40–2.04, p < 0.00001), (OR 1.40, 95 % CI 1.16–1.70, p = 0.0005), (OR 1.37, 95 % CI 1.06–1.76, p = 0.02), (OR 1.46, 95 % CI 1.22–1.76, p < 0.0001) and (OR 1.66, 95 % CI 1.16–2.38, p = 0.005) respectively. Long-term cardiovascular outcomes were also significantly higher in insulin-treated DM patients.

**Conclusion:**

Insulin treatment in these DM patients was associated with a significantly higher short and long-term adverse cardiovascular outcomes after PCI compared to those DM patients not treated by insulin therapy.

## Background

Insulin therapy in Type 2 Diabetes Mellitus (DM) is normally indicated either when oral hypoglycemic medications do not seem to be effective (uncontrolled blood glucose levels despite the use of oral hypoglycemic agents) or initiated especially when these patients suffer from diabetic complications. However, the effect of insulin therapy on adverse cardiovascular outcomes in these DM patients has been a subject of debate. Several studies have shown that compared to non-insulin treated DM patients, insulin-treated DM patients are associated with many adverse cardiovascular outcomes after Percutaneous Coronary Intervention (PCI). For example, the study conducted by Tada et al. in [1] concluded that an excess risk of serious cardiovascular events was observed in the insulin-treated DM compared to non-insulin treated DM patients after PCI [1]. Another study conducted by Akin et al., and including patients from the German Drug-Eluting Stent (DES.DE) registry revealed that even with Drug-Eluting Stents (DES), the annual risks for death, Target Vessel Revascularization (TVR), and, thrombotic events remained higher in DM patients treated with insulin compared to those without insulin treatment [2]. However, other studies showed slightly different results. Results from the study conducted by Kirtane in 2008 showed that rates of stent thrombosis and all-cause mortality were similar among DM patients treated with DES and Bare Metal Stents (BMS) irrespective of insulin-treated or non-insulin treated status. The author also precise that there were no differences in the 4-year composite rates of death or myocardial infarction (MI), death or Q-wave MI, or, cardiac death or MI between paclitaxel eluting stents and BMS in these DM patients with insulin or non-insulin treatment [3]. Therefore, in order to confirm whether or not, insulin-treated DM patients have more adverse outcomes than non-insulin treated DM patients, we sought to compare the short-term and long-term adverse cardiovascular outcomes between insulin-treated and non-insulin treated DM patients after PCI.

## Methods

### Data sources and search strategy

PubMed and Embase were searched for Randomized Controlled Trials (RCTs) and observational studies by typing the words or phrases ‘diabetes and percutaneous coronary intervention/PCI’ or ‘insulin-treated and non-insulin treated diabetes mellitus and PCI’. To further enhance this search, the term ‘angioplasty’ has also been used. All references of relevant studies were also reviewed for relevant articles. No language restriction was applied.

### Inclusion and exclusion criteria

Studies were included if:

- They were RCTs or observational studies dealing with insulin-treated and non-insulin treated DM patients after PCI irrespective of the types of stents implanted.
- Adverse cardiovascular outcomes were reported in these DM patients.
- They had either a short-term follow up period (<1 year) or a long-term follow-up period of ≥1 year after PCI.

Studies were excluded if:

- Adverse clinical outcomes were not among the clinical endpoints.
- They were meta-analyses, case studies or letter to editors.
- No control group/non-insulin treated DM patients were absent.
- They did not include data with discontinuous variables or data which could be easily converted to discontinuous variables.
- Duplicates.

### Definitions, outcomes and follow up periods

*Diabetic patients* referred to as Type 2 DM patients, were defined as patients with a fasting blood glucose (FBG) level of >7.0 mmol/L or with oral glucose tolerance test (OGTT) level of >11.1 mmol/L at least on two separate occasions. In this study, DM patients were divided into insulin-treated and non-insulin treated DM patients.

Insulin-treated/insulin-dependent DM patients were those who required insulin therapy while non-insulin treated/non-insulin dependent DM patients were those patients who required or did not require oral hypoglycemic agents but did not receive insulin therapy.

### The adverse cardiovascular outcomes were

- *Death:* defined as all-cause mortality including cardiac and non-cardiac mortality. If death was not clearly defined whether it was cardiac or non-cardiac or both, we have assumed it to be death of all causes and have used the data in our study.
- *Major adverse cardiac effects (MACEs):* were defined as death of cardiac or procedure-related origin, MI, and/or, revascularization after stents implantation. Since in only a few studies, data for major adverse cardiac and cerebrovascular events (MACCEs) have been given, we have considered MACEs and MACCEs to be in the same category.
- *Target lesion revascularization (TLR) and Target vessel revascularization (TVR):* TLR was defined as clinically indicated percutaneous or surgical revascularization of the index lesion and TVR concerned the vessel affected. Revascularization was clinically indicated if there was >70 % diameter stenosis on angiography or >50 % stenosis together with a positive stress test or ischemic symptoms.
- *Myocardial infarction (MI):* was defined as re-infarction which occurred in these diabetic patients after PCI. It could be Q-wave and non-Q wave MI together, STEMI and NSTEMI together, fatal and non-fatal MI or, any of them depending on which one was listed in the studies we have included in this meta-analysis. If data concerning only non-fatal MI was available, we have omitted and excluded them from our study.
- *Stent thrombosis:* Any type of stent thrombosis including definite and probable stent thrombosis as well as subacute stent thrombosis have been considered in this study.

*Short term follow-up period* was defined as a follow-up period of <1 year. In-hospital follow up has also been included in this short-term follow up period. A follow-up period of up to 12 months or follow up during a whole 1 year period was also considered as short term follow-up.

*Long term follow-up period* was defined as a follow up at 1 year or more (≥1 year).

### Data extraction and quality assessment

Two authors (P.K.B and N.L) independently reviewed the data and assessed the eligibility and methodological quality of each eligible trial. Information regarding study and patient characteristics, intervention strategies, and the pre-specified clinical outcomes was systematically extracted. Disagreements were discussed between the authors, and if the authors could not reach a consensus, disagreements were resolved by the third author (M.H.C). The bias risk of trials was assessed with the components recommended by the Cochrane Collaboration, including sequence generation of the allocation, allocation concealment, blinding of participants, personnel, outcome assessors, incomplete outcome data, selective outcome reporting, and, other sources of bias [4].

### Methodological quality and statistical analysis

Study selection, data collection, analysis, and reporting of the results were performed using the recommendations of the PRISMA (Preferred Reporting Items for Systematic Reviews and Meta-Analyses) statement [5]. Heterogeneity across trials was assessed using the Cochrane Q-statistic (p < 0.05 was considered significant) and I^2^-statistic. I^2^ describes the percentage of total variation across studies; that is, due to heterogeneity rather than chance. A value of 0 % indicates no heterogeneity, and larger values indicate increased heterogeneity. If I^2^ was <50 %, fixed effect model was used. However, if I^2^ was >50 %, a random effect model was used. Publication bias was visually estimated by assessing funnel plots. We calculated odd ratios (OR) and 95 % confidence intervals (CIs) for categorical variables. The pooled analyses were performed with RevMan 5.3 software.

#### Ethics

Ethical approval was not necessary as this study is a Systematic Review and Meta-Analysis.

## Results

### Study selection

**Fig. 1:**
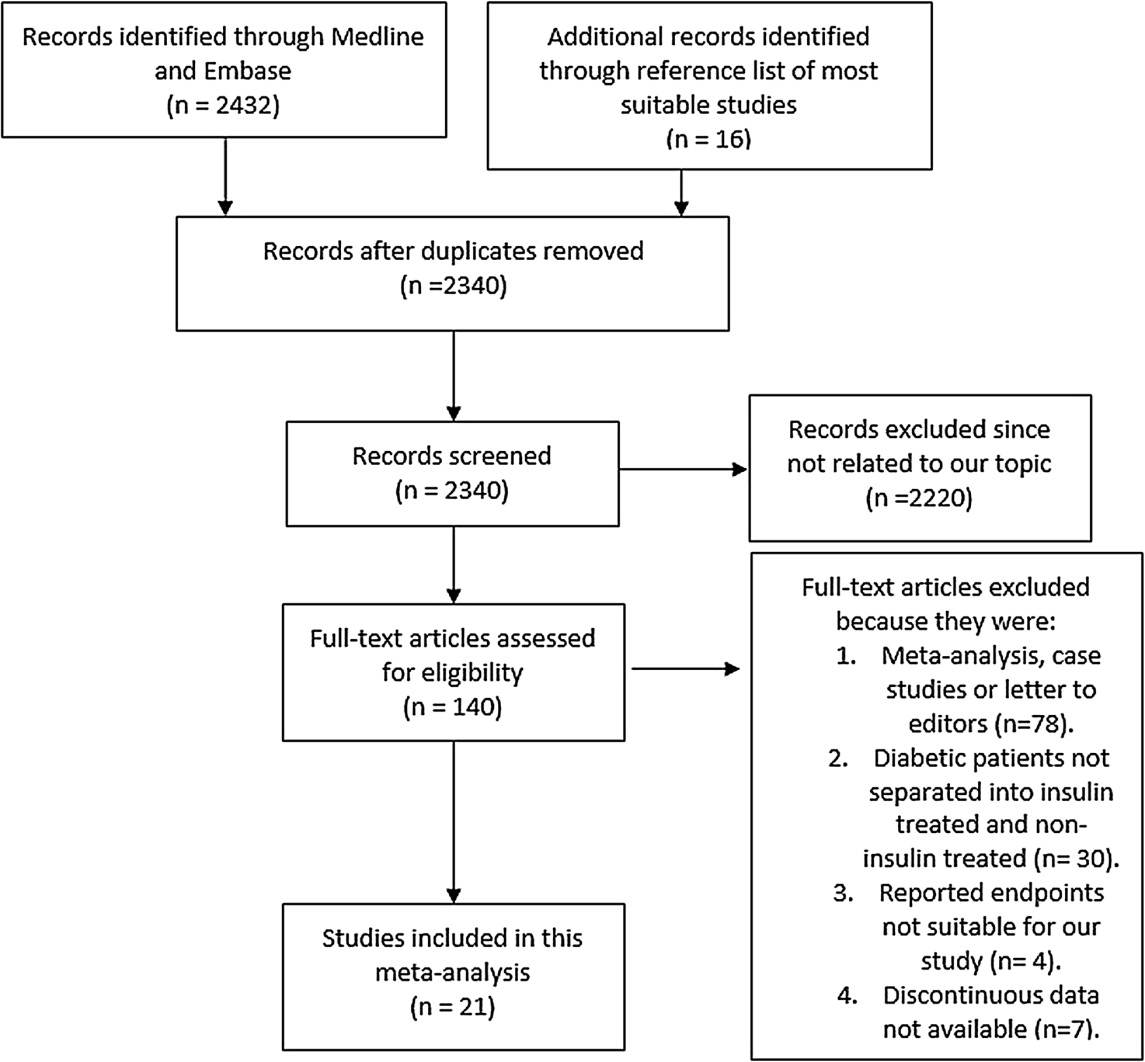
The flow diagram of study selection

2432 articles were identified by title and abstract. 16 additional articles were identified from reference lists of appropriate studies. After elimination of duplicates, 2340 articles were further screened. 2220 articles were excluded since they were not related to the title of our study. 140 full-text articles were finally assessed for eligibility of which, 119 were further excluded for several reasons: they were meta-analyses, case studies or letters to editor, insulin-treated and non-insulin treated diabetics were not separated into 2 different groups for comparison, they did not report the correct endpoints for our study or discontinuous data were not provided. Finally 21 studies have been selected and included in this meta-analysis. The flow diagram for this study selection has been shown in Fig. 1.

### Baseline characteristics

These 21 studies which have been included in this systematic review and meta-analysis consisted of a total of 21,759 DM patients including 6250 insulin-treated and 15,509 non-insulin treated patients. The baseline features of each included study have been shown in Table 1.

**Table 1:** shows the baseline characteristics of each included study

Studies	Age (year)	Male (%)	Ht (%)	Ds (%)	Cs (%)
	IT/NIT	IT/NIT	IT/NIT	IT/NIT	IT/NIT
Abizaid [30]	63.0/63.0	49.5/63.6	73.3/67.5	60.0/64.0	48.9/48.6
Akin [2]	66.9/66.6	65.4/75.0	92.4/92.6	80.7/83.5	14.9/19.3
Antoniucci [31]	69.0/68.0	65.0/73.0	40.0/43.0	30.0/30.0	17.0/21.0
Dangas [8]	62.6/63.2	61.3/76.5	87.5/83.2	–	17.9/14.7
Hermillier [32]	62.2/62.2	63.5/63.5	81.1/81.1	71.4/71.4	–
Jain [33]	66.6/64.9	62.2/71.8	82.1/77.5	67.9/67.7	13.9/18.0
Kereiakes [34]	63.3/63.3	63.3/63.3	87.0/87.0	82.5/82.5	18.3/18.3
Kirtane [3]	63.0/63.0	64.7/64.7	82.1/82.1	74.0/74.0	18.4/18.4
Kirtane [16]	64.0/64.0	60.4/60.4	90.6/90.6	87.1/87.1	54.1/54.1
Kuchulakanti [35]	65.1/65.1	60.5/60.5	89.0/89.0	88.5/88.5	16.0/16.0
Kumar [15]	62.0/67.0	62.0/67.0	94.0/93.0	89.0/92.0	11.0/8.0
Mehran [36]	63.0/66.0	52.0/61.0	77.0/77.0	71.0/67.0	11.0/12.0
Mulukutla [37]	63.5/64.0	50.7/61.5	84.8/83.1	79.5/77.3	16.9/19.4
Nakamura [38]	66.2/67.2	66.2/75.4	68.1/72.0	58.0/60.4	12.1/19.5
Schofer [39]	60.0/62.0	71.0/77.0	73.0/75.0	65.0/72.0	13.0/20.0
Stein [40]	58.0/60.0	53.1/66.1	56.8/63.0	–	–
Stone [41]	63.8/63.8	63.2/63.2	83.1/83.1	79.4/79.4	19.6/19.6
Tada [1]	66.7/67.9	67.0/76.0	76.0/78.0	–	16.0/21.0
Witzenbichler [42]	64.5/64.5	73.4/73.4	72.3/72.3	60.3/60.3	56.8/56.8
Kappetein [43]	65.4/65.4	71.0/71.0	70.0/70.0	82.0/82.0	16.0/16.0

*IT* insulin-treated diabetics, *NIT* non-insulin treated diabetics, *Ht* hypertension, *Ds* dyslipidemia, *Cs* current smoker

Dyslipidemia included abnormal lipid or cholesterol level or treated hyperlipidemia depending of which data have been given in the studies.

A good quality of this meta-analysis is that the studies included were mainly articles published in highly qualified Journals such as the Journal of American College of Cardiology, the Journal of Circulation, the American Heart Association and the American Journal of Cardiology.

According to the baseline characteristics, no significant differences have been found between the two groups.

The number of insulin-treated and non-insulin treated DM patients as well as their corresponding follow up periods have been given in Table 2.

**Table 2:** Number of insulin-treated and non-insulin treated patients with their corresponding follow up periods

Included studies	Insulin-treated DM (n)	Non-insulin treated DM (n)	Follow-up period
Abizaid [30]	97	151	During 1 year
Akin [2]	581	1078	During 1 year
Antoniucci [31]	84	82	6 months
Dangas [8]	325	631	1 month, 5 years
Hermillier [32]	105	213	At 1 year
Jain [33]	644	1919	During 1 year
Kereiakes [34]	314	826	At 1 year
Kirtane [3]	265	562	4 years
Kirtane [16]	137	319	At 1 year
Kuchulakanti [35]	265	586	6 months
Kumar [15]	115	182	9 months
Mehran [36]	81	114	In-hospital
Moussa [18]	82	197	9 months
Mulukutla [37]	817	1749	During 1 year
Nakamura [38]	200	647	At 3 years
Schofer [39]	48	117	6 months
Stein [40]	352	781	In hospital
Stone [41]	494	1375	2 years
Tada [1]	996	3404	3 years
Witzenbichler [42]	159	434	At 1 year
Kappetein [43]	89	142	5 years

*DM* diabetes mellitus

According to Table 2, 12 studies had a short-term follow up period whereas 10 studies had a long-term follow up period after PCI.

### Main results of this meta-analysis

**Fig. 2:**
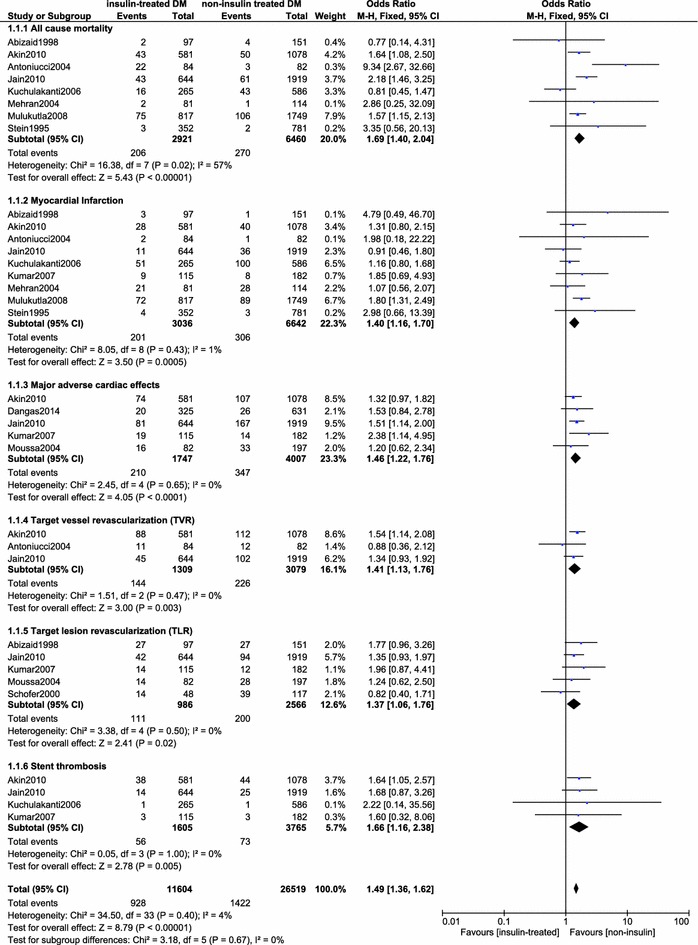
Forest plot comparing the short term cardiovascular outcomes between insulin-treated and non-insulin treated diabetic patients after PCI

The results of this meta-analysis showed that during this short-term follow up period (<1 year), insulin-treated DM patients had significantly higher cardiovascular outcomes: All-cause mortality (OR 1.69, 95 % CI 1.40–2.04, p < 0.00001), MI (OR 1.40, 95 % CI 1.16–1.70, p = 0.0005), TLR (OR 1.37, 95 % CI 1.06–1.76, p = 0.02), TVR (OR 1.41, 95 % CI 1.13–1.76, p = 0.003), MACEs (OR 1.46, 95 % CI 1.22–1.76, p < 0.0001) and, Stent thrombosis (OR 1.66, 95 % CI 1.16–2.38, p = 0.005) compared to non-insulin treated DM patients after PCI. The results for the short-term outcomes have been illustrated in Fig. 2.

**Fig. 3:**
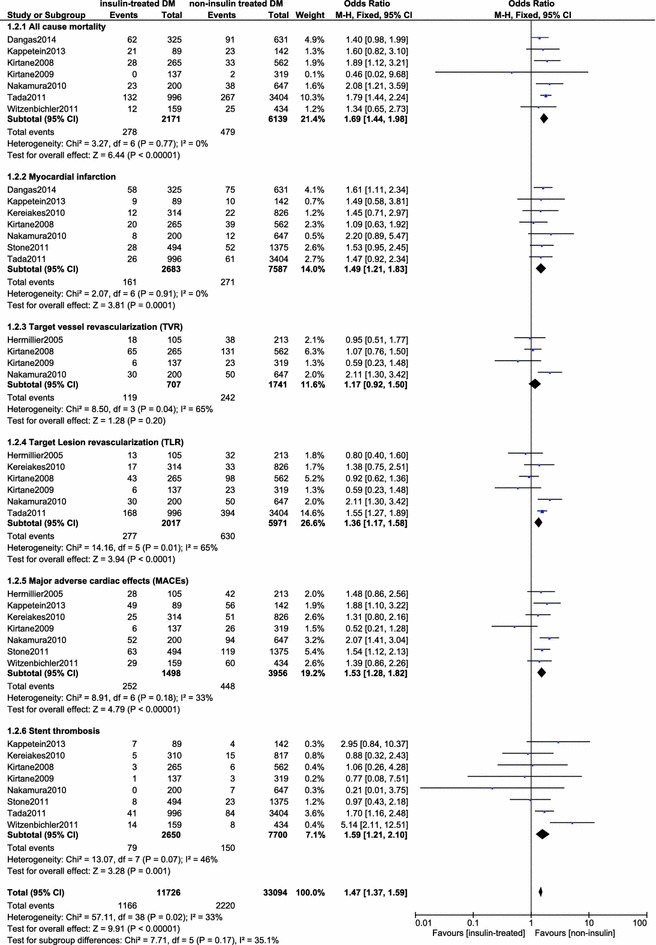
Forest plot comparing the long term cardiovascular outcomes between insulin-treated and non-insulin treated diabetic patients after PCI

During the long-term follow up (≥1 year), the cardiovascular outcomes in insulin-treated DM patients were still significantly higher: All-cause mortality (OR 1.69, 95 % CI 1.44–1.98, p < 0.00001), MI (OR 1.49, 95 % CI 1.21–1.83, p = 0.0001), TLR (OR 1.36, 95 % CI 1.17–1.58, p < 0.0001), MACEs (OR 1.53, 95 % CI 1.28–1.82, p < 0.00001) and, Stent thrombosis (OR 1.59, 95 % CI 1.21–2.10, p = 0.001) compared to non-insulin treated DM patients after PCI. The results for the long term outcomes have been illustrated in Fig. 3.

**Fig. 4:**
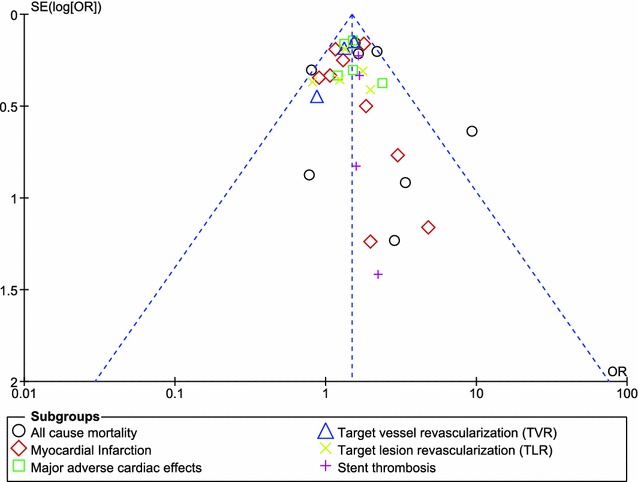
Funnel plot assessing publication bias in the included studies. For all of the above analyses, sensitivity analyses yielded consistent results

For all of the above analyses, sensitivity analyses yielded consistent results. Based on a visual inspection of the funnel plot, there has been no evidence of publication bias for the included studies that assessed all clinical endpoints. The funnel plot has been illustrated in Fig. 4.

## Discussion

### Aim of this study

Type 2 DM patients have worse adverse cardiovascular outcomes after PCI [6, 7]. Insulin therapy is appropriate for those patients in whom oral hypoglycemic drugs are not very effective, and for those type 2 DM patients who suffer from micro-vascular and macro-vascular complications which are late manifestations of this disease. This meta-analysis compares the adverse cardiovascular outcomes between insulin-treated and non-insulin treated DM patients after PCI.

### Results of our study

Results from this meta-analysis show that both, the short-term and long-term adverse cardiovascular outcomes such as mortality, MI, TLR, MACEs and stent thrombosis are significantly higher in insulin-treated DM compared to non-insulin treated DM patients after PCI.

### Possible reasons and explanation

Several reasons have been thought to be responsible for this significantly higher rate of adverse cardiovascular outcomes in these insulin-treated DM patients after PCI. First of all, insulin-treated DM patients have worse clinical outcome regardless of the treatment regimen, which could either be due to more aggressive disease in these patients or an adverse effect of this insulin therapy [8]. Normally, insulin therapy is initiated in a more advanced stage of diabetes. Logistically, a higher rate of adverse outcomes should be expected in these complicated patients after PCI.

In addition, studies have shown that insulin-treated DM patients had higher body mass index, hemoglobin A1c (glycosylated hemoglobin), and, blood urea nitrogen (BUN) levels than non-insulin treated DM patients, and were more likely to have a history of stroke, hypertension, congestive heart failure, and, acute coronary syndrome when compared with non-insulin treated DM patients [8]. Hence, these co-morbidities could be another reason for these increased adverse outcomes in these insulin-treated DM patients.

Moreover, iatrogenic hyperinsulinemia controls hyperglycemia in insulin-treated DM patients but this can also promote pro-inflammatory macrophage responses and stimulate hormonal over-activation of signal transduction pathways, which affect progression of atherogenesis and disturb hemodynamic control and cardiovascular function by disrupting the balanced synthesis and release of endothelial mediators [9–11]. This has been explained in more details below. At the same time, insulin might be a marker of high-risk patients, not only because of more severe insulin resistance but also because of more prolonged diabetes mellitus.

Normally, endogenous hyperinsulinemia of type 2 DM is associated with increased hepatic synthesis of cholesterol and triglycerides [12]. Studies have shown that glucose control in type 1 DM often requires exogenous insulin in amounts far greater than that secreted by normal beta-cells. The relation between hyperinsulinemia and hepatic markers of atherogenesis was investigated by Wang and colleagues in a murine model of type 1 DM [13]. Although insulin injection significantly raised plasma levels of PCSK-9, the rise did not exceed that of nondiabetic mice with lower insulin levels. In contrast, insulin injection appeared to trigger the release of the pro-inflammatory mediators tumor necrosis factor; and interleukin-1; in diabetic mice to levels higher than that seen in non-diabetic mice. The findings suggest that exogenous insulin promotes pro-inflammatory macrophage responses independent of markers of hepatic cholesterol processing [13], consistent with earlier clinical findings of increased inflammatory markers in coronary atherectomy specimens from DM patients [14].

Also, insulin treatment in type 2 DM has been associated with increased platelet aggregation, a finding of particular concern given current controversies about ongoing risk for stent thrombosis after DES implantation [15].

Another reason for this higher rate of adverse cardiovascular outcomes could be a greater prevalence of a family history of coronary artery disease in insulin-treated DM patients and a lesser prevalence of hyperlipidemia in non-insulin treated DM patients shown in the study conducted by Kirtane in 2009 [16].

### Other researches

Similar to this meta-analysis, a study conducted by Claessen in 2011 showed that patients with insulin-treated DM had higher long-term mortality compared to patients with non-insulin treated DM (16.6 vs 11.9 %, p < 0.049) after PCI [17]. Moreover, the (SIRIUS) trial with 131 DM patients receiving Sirolimus-Eluting Stent also supported our results showing a higher MACEs rate (15.8 vs 6.5 %, p < 0.001), and TLR rate (13.2 vs 4.3 %, p < 0.001 in patients requiring insulin compared to those who did not require insulin. In the Taxus-IV trial of a paclitaxel-eluting stent, higher rates of overall MACEs were observed in insulin-treated compared to non–insulin-treated DM patients [18].

A study by Daemen et al. [19] published in 2007 showed that all-cause mortality was higher in insulin-treated DM patients (16.7 vs 9.6 %, p < 0.013) compared to those without insulin therapy. However, this study found no differences in TLR between these insulin and non-insulin treated DM patients [19].

Several studies have reported results which were different from our meta-analysis too. Insulin therapy may not always be associated with adverse cardiovascular events. Recently, several researches have been published on insulin resistance. The study by Trifunovic et al. showed that insulin resistance assessed by the Homeostasis Model Assessment (HOMA) index during the acute phase of the first anterior STEMI in patients without diabetes treated by primary PCI is independently associated with poorer myocardial reperfusion, impaired coronary microcirculatory function, and potentially with larger final infarct size [20]. Another study published by Iguchi T et al. suggested that insulin resistance might be associated with coronary plaque vulnerability [21]. Moreover, the study by Lopez-de-Andres et al. showed that higher comorbidity and female gender are associated with a higher in-hospital mortality in PCI procedures and in-hospital mortality was higher in patients without diabetes than those with diabetes indicating that maybe insulin therapy is not the real cause of adverse outcomes in these patients [22]. Also, the study published by Kuramitsu et al. in 2013 concluded that post-challenge hyperglycemia is associated with future cardiovascular events in patients with stable angina undergoing PCI [23].

Furthermore, the study by Ong et al. reported results in 293 diabetic patients from the non-concurrent Rapamycin-Eluting Stent Evaluated At Rotterdam Cardiology Hospital (RESEARCH) and Taxus-Stent Evaluated At Rotterdam Cardiology Hospital [T-SEARCH] registries who received either sirolimus- or paclitaxel-eluting stents. Insulin-treated patients had a higher crude rate of MACEs at 1 year compared with other DM patients (27.4 vs 14.6 %, p < 0.008), but the difference was not significant after multivariable adjustment [24].

Another study performed by Berenguer et al. showed higher restenosis rates for insulin-treated DM patients after sirolimus-eluting stenting as well as a non-statistically significant difference for the clinical outcome of target vessel failure (death, MI, or TVR, 17.4 vs 7.7 %, p < 0.07) [25]. These investigators, however, also noted that insulin treatment was not a significant independent predictor of clinical outcome. Of note, these registry studies also had limited power to detect statistical significance with only 72 and 46 insulin-treated patients, respectively.

### Novelty in this study

This meta-analysis compares the cardiovascular outcomes between insulin-treated and non-insulin treated DM patients after PCI. Several meta-analyses comparing BMS and DES in DM patients [26], comparing the effectiveness of different types of DES [27, 28], or comparing the clinical outcomes in DM patients undergoing PCI and Coronary Artery Bypass Grafting (CABG) have been conducted but no one has yet conducted a meta-analysis between insulin-treated and non-insulin treated DMpatients after PCI [29]. Moreover, this meta-analysis which includes 21,759 DM patients from 9 RCTs and 12 observational studies, compares both the short term and long term cardiovascular outcomes in these patients.

### Limitations

First of all, due to the limited study number and population size of insulin-treated DM patients, the power of the analysis might be restricted to some extent. Another limitation could be the short term follow up period. In-hospital outcomes have been included in the short-term follow up category along with follow up during a 1 year period. This could affect the results of this study to an extent. Inclusion of observational studies together with RCTs in this meta-analysis is supposed to reduce the risk for bias. However, this inclusion of observational studies could on the other hand be a limitation in this study.

## Conclusion

Insulin treatment in these DM patients was associated with a significantly higher short and long-term adverse cardiovascular outcomes after PCI compared to those DM patients not treated by insulin therapy. Therefore, compared to non-insulin treated DM patients, the prognosis in insulin-treated DM patients is not so good after PCI.

## Abbreviations

DM: diabetes mellitus
PCI: percutaneous coronary intervention
MACEs: major adverse cardiac effects
MI: myocardial infarction
TVR: target vessel revascularization
TLR: target lesion revascularization
DES: drug eluting stents

## References

[1] Tada T, Kimura T, Morimoto T, Ono K, Furukawa Y, Nakagawa Y (2011). Comparison of 3 year clinical outcomes after sirolimus eluting stent implantation among insulin-treateddiabetic, non-insulin-treated diabetic, and non-diabetic patients from j-Cypher registry. Am J Cardiol.

[2] Akin I, Bufe A, Eckardt L, Reinecke H, Senges J, Richardt G (2010). Comparison of outcomes in patients with insulin-dependent versus non-insulin dependent diabetes mellitusreceiving drug-eluting stents (from the first phase of the prospective multicenter German DES.DE registry). Am J Cardiol.

[3] Kirtane AJ, Ellis SG, Dawkins KD, Colombo A, Grube E, Popma JJ (2008). Paclitaxel-eluting coronary stents in patients with diabetes mellitus: pooled analysis from 5 randomized trials. J Am Coll Cardiol.

[4] Higgins JPT, Altman DG. Assessing risk of bias in included studies. In: Higgins JPT, Green S, eds. Cochrane handbook for systematic reviews of interventions. Wiley, 2008:187-241.

[5] Liberati A, Altman DG, Tetzlaff J, Mulrow C, Gøtzsche PC, Ioannidis JP (2009). The PRISMA statement for reporting systematic reviews and meta-analyses of studies that evaluate healthcareinterventions: explanation and elaboration. BMJ.

[6] Elezi S, Kastrati A, Pache J, Wehinger A, Hadamitzky M, Dirschinger J (1998). Diabetes mellitus and the clinical and angiographic outcome after coronary stent placement. J Am Coll Cardiol.

[7] Mathew V, Gersh BJ, Williams BA, Laskey WK, Willerson JT, Tilbury RT (2004). Outcomes in patients with diabetes mellitus undergoing percutaneous coronary intervention in the current era: a report from the Prevention of REStenosis with Tranilast and its Outcomes (PRESTO) trial. Circulation.

[8] Dangas GD, Farkouh ME, Sleeper LA, Yang M, Schoos MM, Macaya C (2014). Long-term outcome of PCI versus CABG in insulin and non-insulin-treated diabetic patients: results from the FREEDOM trial. J Am Coll Cardiol.

[9] Muniyappa R, Montagnani M, Koh KK, Quon MJ (2007). Cardiovascular actions of insulin. Endocr Rev.

[10] Potenza MA, Addabbo F, Montagnani M (2009). Vascular actions of insulin with implications for endothelial dysfunction. Am J Physiol Endocrinol Metab.

[11] Wang MY, Yu X, Lee Y (2013). Iatrogenic hyperinsulinemia in type 1 diabetes: its effect on atherogenic risk markers. J Diabetes Compl.

[12] Unger RH, Orci L (2010). Paracrinology of islets and the paracrinopathy of diabetes. Proc Natl Acad Sci USA.

[13] Wang M-Y, Yu X, Lee L (2009). Iatrogenic hyperinsulinemia in type 1 diabetes: its effect on atherogenic risk markers. J Diabetes Complications.

[14] Moreno PR, Murcia AM, Palacios I, Leon MN, Bernardi VH, Fuster V (2002). Coronary composition and macrophage infiltation in atherectomy specimens from patients with diabetes mellitus. Circulation.

[15] Kumar R, Lee TT, Jeremias A, Ruisi CP, Sylvia B, Magallon J (2007). Comparison of outcomes using sirolimus-eluting stenting in diabetic versus nondiabetic patients with comparison of insulin versus non-insulin therapy in the diabetic patients. Am J Cardiol.

[16] Kirtane AJ, Patel R, O’Shaughnessy C, Overlie P, McLaurin B, Solomon S (2009). Clinical and angiographic outcomes in diabetics from the ENDEAVOR IV trial: randomized comparison of zotarolimus- and paclitaxel-eluting stents in patients with coronary artery disease. JACC Cardiovasc Interv.

[17] Claessen BE, Dangas GD, Godino C, Lee SW, Obunai K, Carlino M (2011). Long-term clinical outcomes of percutaneous coronary intervention for chronic total occlusions in patients withversus without diabetes mellitus. Am J Cardiol.

[18] Moussa I, Leon MB, Baim DS, O’Neill WW, Popma JJ, Buchbinder M (2004). Impact of sirolimus-eluting stents on outcome in diabetic patients: a SIRIUS (SIRolImUS-coated Bx Velocity balloon-expandable stent in the treatment of patients with de novo coronary artery lesions) substudy. Circulation.

[19] Daemen J, Garcia-Garcia HM, Kukreja N, Imani F, de Jaegere PP, Sianos G (2007). The long-term value of sirolimus- and paclitaxel-eluting stents over bare metal stents in patients with diabetes mellitus. Eur Heart J.

[20] Trifunovic D (2014). Acute insulin resistance in ST-segment elevation myocardial infarction in non-diabetic patients is associated with incomplete myocardial reperfusion and impaired coronary microcirculatory function. Cardiovasc Diabetol.

[21] Iguchi T (2014). Insulin resistance is associated with coronary plaque vulnerability: insight from optical coherence tomography analysis. Eur Heart J Cardiovasc Imaging.

[22] Lopez-de-Andres A (2014). National trends in utilization and outcomes of coronary revascularization procedures among people with and without type 2 diabetes in Spain (2001–2011). Cardiovasc Diabetol.

[23] Kuramitsu S (2013). Impact of post-challenge hyperglycemia on clinical outcomes in Japanese patients with stable angina undergoing percutaneous coronary intervention. Cardiovasc Diabetol.

[24] Ong AT, Aoki J, van Mieghem CA, Rodriguez Granillo GA, Valgimigli M, Tsuchida K (2005). Comparison of short- (1 month) and long- (12 months) term outcomes of sirolimus- versus paclitaxel-eluting stents in 293 consecutive patients with diabetes mellitus (from the RESEARCH and T-SEARCH registries). Am J Cardiol.

[25] Berenguer A, Mainar V, Bordes P, Valencia J, Gomez S (2006). Efficacy of sirolimus-eluting stents in diabetics with complex coronary lesions. Rev Esp Cardiol.

[26] De Luca G, Dirksen MT, Spaulding C, Kelbæk H, Schalij M, Thuesen L (2013). Meta-analysis comparing efficacy and safety of first generation drug-eluting stents to bare-metal stents in patients with diabetes mellitus undergoing primary percutaneous coronary intervention. Am J Cardiol.

[27] Liu Y, Gao L, Song Y, Chen L, Xue Q, Tian J, Wang Y, Chen Y (2015). Efficacy and safety of limus-eluting versus paclitaxel-eluting coronary artery stents in patients with diabetes mellitus: a meta-analysis. Int J Cardiol.

[28] Bangalore S, Kumar S, Fusaro M, Amoroso N, Kirtane AJ, Byrne RA (2012). Outcomes with various drug eluting or bare metal stents in patients with diabetes mellitus: mixed treatment comparison analysis of 22,844 patient years of follow-up from randomised trials. BMJ.

[29] Verma S, Farkouh ME, Yanagawa B, Fitchett DH, Ahsan MR, Ruel M (2013). Comparison of coronary artery bypass surgery and percutaneous coronary intervention in patients with diabetes: a meta-analysis of randomised controlled trials. Lancet Diabetes Endocrinol.

[30] Abizaid A, Kornowski R, Mintz GS, Hong MK, Abizaid AS, Mehran R (1998). The influence of diabetes mellitus on acute and late clinical outcomes following coronary stent implantation. J Am Coll Cardiol.

[31] Antoniucci D, Valenti R, Migliorini A, Parodi G, Moschi G, Memisha G (2004). Impact of insulin requiring diabetes mellitus on effectiveness of reperfusion and outcome of patients undergoing primary percutaneous coronary intervention for acute myocardial infarction. Am J Cardiol.

[32] Hermiller JB, Raizner A, Cannon L, Gurbel PA, Kutcher MA, Wong SC (2005). Outcomes with the polymer-based paclitaxel-eluting TAXUS stent in patients with diabetes mellitus: the TAXUS-IV trial. J Am Coll Cardiol.

[33] Jain AK, Lotan C, Meredith IT, Feres F, Zambahari R, Sinha N (2010). Twelve- month outcomes in patients with diabetes implanted with a zotarolimus-eluting stent: results from the E-Five Registry. Heart.

[34] Kereiakes DJ, Cutlip DE, Applegate RJ, Wang J, Yaqub M, Sood P (2010). Outcomes in diabetic and nondiabetic patients treated with everolimus- or paclitaxel-eluting stents: results from the SPIRIT IV clinical trial (Clinical Evaluation of the XIENCE V Everolimus Eluting Coronary Stent System). J Am Coll Cardiol.

[35] Kuchulakanti PK, Chu WW, Torguson R, Clavijo L, Wolfram R, Mishra S (2006). Sirolimus-eluting stents versus Paclitaxel-eluting stents in the treatment of coronary artery disease in patientswith diabetes mellitus. Am J Cardiol.

[36] Mehran R, Dangas GD, Kobayashi Y, Lansky AJ, Mintz GS, Aymong ED (2004). Short- and long-term results after multivessel stenting in diabetic patients. J Am Coll Cardiol.

[37] Mulukutla SR, Vlachos HA, Marroquin OC, Selzer F, Holper EM, Abbott JD (2008). Impact of drug-eluting stents among insulin-treated diabetic patients: a report from the National Heart, Lung, and Blood Institute Dynamic Registry. JACC Cardiovasc Interv.

[38] Nakamura M, Yokoi H, Hamazaki Y, Watarai M, Kijima M, Mitsudo K (2010). Cypher J-PMS Investigators. Impact of insulin-treated diabetes and hemodialysis on longterm clinical outcomes following sirolimus-elutingstent deployment. Insights from a sub-study of the Cypher Stent Japan Post-Marketing Surveillance (Cypher J-PMS) Registry. Circ J.

[39] Schofer J, Schlüter M, Rau T, Hammer F, Haag N, Mathey DG (2000). Influence of treatment modality on angiographic outcome after coronary stenting in diabetic patients: a controlled study. J Am Coll Cardiol.

[40] Stein B, Weintraub WS, Gebhart SP, Cohen-Bernstein CL, Grosswald R, Liberman HA (1995). Influence of diabetes mellitus on early and late outcome after percutaneous transluminal coronary angioplasty. Circulation.

[41] Stone GW, Kedhi E, Kereiakes DJ, Parise H, Fahy M, Serruys PW, Smits PC (2011). Differential clinical responses to everolimus-eluting and Paclitaxel-eluting coronary stents in patients with and without diabetes mellitus. Circulation.

[42] Witzenbichler B, Mehran R, Guagliumi G, Dudek D, Huber K, Kornowski R (2011). Impact of diabetes mellitus on the safety and effectiveness of bivalirudin in patients with acute myocardialinfarction undergoing primary angioplasty: analysis from the HORIZONS-AMI (Harmonizing Outcomes with RevasculariZatiON and Stents in Acute Myocardial Infarction) trial. JACC Cardiovasc Interv..

[43] Kappetein AP, Head SJ, Morice M-C (2013). Treatment of complex coronary artery disease in patients with diabetes: 5-year results comparing outcomes of bypass surgery and percutaneous coronary intervention in the SYNTAX trial. Eur J Cardiothorac Surg.

